# Association between somatic microsatellite instability, hypermutation status, and specific T cell subsets in colorectal cancer tumors

**DOI:** 10.3389/fimmu.2024.1505896

**Published:** 2024-12-23

**Authors:** Claire E. Thomas, Yasutoshi Takashima, Evertine Wesselink, Tomotaka Ugai, Robert S. Steinfelder, Daniel D. Buchanan, Conghui Qu, Li Hsu, Andressa Dias Costa, Steven Gallinger, Robert C. Grant, Jeroen R. Huyghe, Sushma S. Thomas, Shuji Ogino, Amanda I. Phipps, Jonathan A. Nowak, Ulrike Peters

**Affiliations:** ^1^ Public Health Sciences Division, Fred Hutchinson Cancer Center, Seattle, WA, United States; ^2^ Department of Medical Oncology, Dana-Farber Cancer Institute, Boston, MA, United States; ^3^ Division of Human Nutrition and Health, Wageningen University & Research, Wageningen, Netherlands; ^4^ Program in Molecular Pathological Epidemiology (MPE), Department of Pathology, Brigham and Women’s Hospital and Harvard Medical School, Boston, MA, United States; ^5^ Department of Epidemiology, Harvard T.H. Chan School of Public Health, Boston, MA, United States; ^6^ Cancer Epidemiology Program, Dana-Farber/Harvard Cancer Center, Boston, MA, United States; ^7^ Colorectal Oncogenomics Group, Department of Clinical Pathology, Melbourne Medical School, The University of Melbourne, Parkville, VIC, Australia; ^8^ University of Melbourne Centre for Cancer Research, The University of Melbourne, Parkville, VIC, Australia; ^9^ Genomic Medicine and Family Cancer Clinic, The Royal Melbourne Hospital, Parkville, VIC, Australia; ^10^ Department of Biostatistics, University of Washington, Seattle, WA, United States; ^11^ Department of Medical Oncology, Dana-Farber Cancer Institute and Harvard Medical School, Boston, MA, United States; ^12^ Lunenfeld-Tanenbaum Research Institute, Sinai Health System, Toronto, ON, Canada; ^13^ Division of Medical Oncology and Hematology, Princess Margaret Cancer Centre, University Health Network, Toronto, ON, Canada; ^14^ Broad Institute of Massachusetts Institute of Technology (MIT) and Harvard, Cambridge, MA, United States; ^15^ Institute of Science Tokyo, Tokyo, Japan; ^16^ Department of Epidemiology, University of Washington, Seattle, WA, United States; ^17^ Department of Pathology, Dana-Farber Cancer Institute, Boston, MA, United States

**Keywords:** microsatellite instability, hypermutation, DNA mismatch repair, T cells, epithelial, stromal, colorectal cancer, molecular epidemiology

## Abstract

**Background:**

Microsatellite instability-high (MSI-high) tumors comprise ~15% of sporadic colorectal cancers (CRC) and are associated with elevated T cell infiltration. However, the universality of this response across T cell subtypes with distinct functions is unknown.

**Methods:**

Including 1,236 CRC tumors from three observational studies, we conducted *in-situ* T cell profiling using a customized 9-plex (CD3, CD4, CD8, CD45RA, CD45RO, FOXP3, KRT, MKI67, and DAPI) multispectral immunofluorescence assay. MSI status was assessed through polymerase chain reaction or immunohistochemical assays. We used multivariable ordinal logistic regression to estimate odds ratios (OR per increasing quantile) and 95% confidence intervals (CIs) for the association of MSI status with quantiles of T cell densities in either tumor epithelial or stromal tissue areas.

**Results:**

Compared to microsatellite instability low or microsatellite stable (MSI-low/MSS) tumors, MSI-high status was associated with higher density for the majority of immune subsets (twelve out of eighteen) in both epithelial and stromal tissue areas. The strongest associations were for CD3^+^CD8^+^ T cells in epithelial areas [OR (95% CI) for naive, memory, and regulatory subsets = 3.49 (2.57, 4.75); 2.82 (2.10, 3.78); 3.04 (2.24, 4.13), respectively]. Conversely, stromal area CD3^+^CD4^+^ memory T cells were inversely associated [OR (95% CI) = 0.68 (0.51, 0.91)].

**Discussion:**

MSI-high status was strongly associated with higher densities of most T cell subsets in both epithelial and stromal tissue areas. Our investigation supports efforts to identify patients who may be more likely to respond to current immunotherapy treatments.

**Significance:**

This study helps us better understand how a clinically relevant tumor phenotype, microsatellite instability status, is related to different functioning T cell densities in colorectal tumors, which may impact future immunotherapy strategies.

## Introduction

Colorectal cancer (CRC) is a heterogeneous disease with different underlying mechanisms leading to different molecular subtypes with varying treatment responses (1). Around 15% of sporadic CRCs are classified as microsatellite instability-high (MSI-high) or DNA mismatch repair-deficient (dMMR); these tumors accumulate a higher burden of somatic mutations, high antigen burden, and high infiltration of T cell lymphocytes overall (2, 3). Given their characteristic high infiltration of T cells, MSI-high tumors are more likely to respond to immune checkpoint inhibitors (ICIs), which has resulted in substantial, durable improvement in treatment of MSI-high CRC (4–6). MSI-high status is strongly correlated with hypermutation status, where around 16% of CRCs are hypermutated, three quarters of which are MSI-high (7, 8). Hypermutation can be caused by multiple factors, including MMR deficiency related to MSI as well as *POLE* mutations that can lead to a hypermutated phenotype without MSI (9).

Although previous studies have shown MSI-high status to be associated with T cell infiltration in CRC (10–13), specifically CD45RO^+^ T cells (14), characterization of infiltrating T cells in these studies has been limited. Specifically, many studies have measured T cell densities using single-plex immunohistochemistry assays to assess individual cell markers, which fails to represent the complexity of T cell response and diverse T cell subsets. A more granular understanding of specific T cell subsets that are associated with the MSI pathway, a strong determinant of prognosis and treatment, may help inform targeted immunotherapy treatment decision-making for both the MSI-high and MSI-low/MSS (microsatellite stable) CRC subtypes.

Utilizing the resources of studies participating in the Genetics and Epidemiology of Colorectal Cancer Consortium (GECCO), we aimed to characterize T-cell response in diverse subsets of CRC tumors using a multiplex immunofluorescence panel and identify which specific T cell subsets, in either epithelial or stromal tumor area regions, are associated with MSI-high and hypermutation status.

## Methods

### Study population

This study was conducted within a subset of studies participating in the Genetics and Epidemiology of Colorectal Cancer Consortium (GECCO) for which tumor immune profiling data were available, including: the Ontario Family Colon Cancer Registry (OFCCR) (15), the Nurses’ Health Study (NHS) (16), and the Health Professionals Follow-up Study (HPFS) (16). The NHS and HPFS cohorts were the basis of the Prospective Cohort Incident Tumor Biobank (PCITB) (17, 18). Data on tumor tissue analyses and other associated metadata constituted the entirety of the PCITB (17, 18). After excluding participants with tumor immune profiling data who did not have data on MSI status available (N=79), data from a total of N=1,236 individuals was available for analysis. The GECCO consortium is an international collaboration that focuses on the identification and characterization of genetic risk factors and gene-environment interactions for CRC and investigates tumor genome/characteristics, microbiome, and immune response (19, 20). Clinical and epidemiologic data were collected by each study through self-reported structured questionnaires or in-person interviews. All participants gave written informed consent and studies were approved by their respective Institutional Review Boards. Studies identified incident CRC either via self-report of diagnosis from study participants, with confirmation via adjudication of medical records (NHS, HPFS), or via population-based cancer registries, regional hospitals, or healthcare management organizations (OFCCR).

### Microsatellite instability (MSI) and hypermutation status calling

Microsatellite instability (MSI) status information was collected by each study according to individual study protocols. The harmonization procedures as well as the methods for individual studies have been previously described (15, 21–26). NHS and HPFS (24) used polymerase chain reaction (PCR) based assessment of MSI status, while OFCCR (15, 25) utilized a combination of PCR-based and immunohistochemical (IHC) assays for MSI. For all studies, tumors were classified as MSI-high if 30% or more of the markers showed instability and non MSI-high if < 30% and > 0% showed instability (MSI-low), or if no marker exhibited instability (microsatellite stable, MSS). To harmonize markers across all studies, we created two categories for downstream analyses, MSI-high and non MSI-high (MSI-low/MSS).

Previously collected targeted sequencing data was available for a subset of participants within the present study to quantify hypermutation status (N=639). Briefly, tumors from OFCCR were sequenced with a 1.34 megabase (Mb) targeted panel covering 205 genes (27), and tumors from NHS and HPFS were sequenced with a 1.96Mb targeted panel expanding the panel to 298 genes. DNA was extracted from formalin-fixed paraffin-embedded (FFPE) CRC tissue that was macrodissected from tissue slides. Matching normal DNA was primarily extracted from either adjacent normal colonic FFPE tissue or peripheral blood. Hypermutation status was defined by plotting point mutations for all samples within each targeted sequencing batch, where two distinct peaks were observed. The minimum value between peaks was used as a cut-point in each sequenced dataset, which were 23 and 26 point mutations, respectively (27).

### T cell profiling

We profiled the *in-situ* T cell landscape of CRC using a multiplexed immunofluorescence (mIF) panel. Tissue microarray (TMA) construction using standard methods has been described in detail elsewhere (28). In brief, core selection from formalin-fixed paraffin-embedded (FFPE) blocks was guided by pathologist review of H&E-stained slides. For the vast majority of CRC cases, 2-4 cores (approximately 0.6 mm) from tumor areas were placed into a TMA block. For a small number of participants only a single core was available due to tissue detachment before and/or after the staining process. TMAs were mounted on negatively charged slides, which generally provide good tissue section adherence. T cell immune microenvironment was assessed for each tumor histologically using a multispectral imaging platform (PhenoImager HT, Akoya Biosciences, Marlborough, MA, USA) for mIF, where TMAs were stained with all markers concurrently, imaged, segmented into epithelial and stromal regions as well as individual cells, phenotyped, and finally quantified into counts. TMAs were magnified at 0.5 um/pixel through the PhenoImager HT platform. Assays were conducted on TMAs using a customized 9-plex panel that included antibodies targeting CD3, CD4, CD8, PTPRC (CD45RO, CD45RA), FOXP3, MKI67 (Ki-67; proliferation), as well as a KRT (keratin, pan-cytokeratin) antibody to identify tumor cells and nuclear DAPI (di-(4-amidinophenyl)-1H-indole-6-carboxamidine) stain. We used the Opal multiplex technique that is a well-established method for detecting multiple biomarkers in a single tissue section (29). To assess potential signal interference for each marker, we stained human lymph node tissue and tonsil tissue without cancer metastasis as a control and set up the fluorescence unmixing condition. Additionally, to minimize background fluorescence and signal interference, multiplex immunofluorescence analysis was performed on autofluorescence slides treated with Opal fluorescences, excluding primary antibodies. The antibodies and staining conditions for multiplex immunofluorescence histological analysis are presented in **Supplementary Table 1**. This marker panel was validated against traditional chromogenic immunohistochemistry, where we performed single-plex immunofluorescence for each marker to assess the performance of T-cell-targeted multiplex immunofluorescence. This involved comparing the single immunohistochemical staining of each marker with lymphoid tissue as a reference.

With pathologist supervision, 9-plex digital fluorescence images were processed using supervised machine learning (inForm 2.6.0, Akoya Biosciences, Marlborough, Massachusetts, U.S.) to segment each region of interest into epithelial and stromal tissue areas. The concomitant single-cell-level analysis was performed using R v.4.3.0. (R Foundation for Statistical Computing, Vienna, Austria). T cell densities (cell count per mm^2^) were then quantified within each TMA core, where we were able to identify naïve, memory, and regulatory helper T cells, naïve, memory, and regulatory cytotoxic T cells, double-negative naïve and memory T cells, and CD3^-^ immune cells based on marker co-localization, all within either epithelial or stromal tissue, resulting in 18 unique subsets (**Table 1**).

**Table 1:** T cell subset definitions by marker co-expression.

	CD3	CD4	CD8	CD45RA	CD45RO	FOXP3
**CD3^+^CD4^+^ naïve**	+	+	−	+	−	−
**CD3^+^CD4^+^ memory**	+	+	−	−	+	−
**CD3^+^CD4^+^ regulatory**	+	+	−	+/−	+/−	+
**CD3^+^CD8^+^ naïve**	+	−	+	+	−	−
**CD3^+^CD8^+^ memory**	+	−	+	−	+	−
**CD3^+^CD8^+^ regulatory**	+	−	+	+/−	+/−	+
**CD3^+^CD4^-^CD8^-^ naïve**	+	−	−	+	−	−
**CD3^+^CD4^-^CD8^-^ memory**	+	−	−	−	+	−
**CD3^-^ immune cells**	−	−	−	+/−	+/−	−

CD3+CD4-CD8- referred to as “double negative” T cells in text.

CD3- immune cells are required to be positive for either CD45RA or CD45RO.

### Statistical analysis

For each CRC case, we used the density metrics of each T cell subset in tumor epithelial or stromal tissue areas averaged across multiple TMA cores. Box plots, including median and interquartile ranges, were used to visualize distributions of T cell subset densities. Wilcoxon non-parametric tests were used to assess for significant differences between MSI-high and MSI-low/MSS for each T cell subset. Participant characteristics were described for the total study population and by MSI status using mean and standard deviation for continuous age, or frequencies and percentages for categorical variables. P values were calculated using t-tests for mean differences for age or chi square for frequencies for categorical variables. Scatter plots and Spearman correlation coefficients were used to determine the association between continuous tumor mutational burden (indels and single nucleotide variants) and T cell densities.

We used multivariable ordinal logistic regression or multivariable binary logistic regression to estimate odds ratios (ORs) and 95% confidence intervals (CIs) for the association of MSI or hypermutation status with quantiles of specific T cell densities in CRC, depending on the percentage of zeros within each subset. Ordinal logistic regression either used quartiles or tertiles of T-cell densities as the outcome, where different subsets were assessed for zero-inflation considering both epithelial and stromal tissue, so the same subset used the same categorization in each tissue area (**Table 2**). To further investigate the impact of MSI status on T cell subsets, we stratified our non-overlapping immune cell subsets by proliferation (MK167, Ki-67) status. When categorized additionally by proliferation status, the same categorization was used for each subset in both proliferating and non-proliferating subsets, as well as in both epithelial and stromal tissue areas (**Table 3**). Subsets with less than 25% zero densities were divided into quartiles, subsets with greater than 25% and less than 60% zero densities into tertiles, and greater than 60% zeros were categorized as binary, and therefore used binary logistic regression. Study batch-specific quantile cut points were used to limit potential batch effects across TMAs. Models were adjusted for age, sex, study batch (NHS & HPFS, OFCCR), and cancer site (proximal, distal, rectal, missing). P-values were adjusted for multiple testing using the false discovery rate (FDR) method for 18 independent tests in primary analyses and 36 independent tests when further stratified by proliferation status, where FDR adjusted P values less than 0.05 were considered significant (30). All P values reported are two-sided. All analyses were performed using R, version 4.4.0 (R Foundation for Statistical Computing, Vienna, Austria) software.

**Table 2:** Association between microsatellite instability status and T cell subset densities.

Outcome	N	Density Zeros N (%)	Categorization	OR per increasing quantile (95% CI) for MSI-high compared to MSI-low/MSS^a^	P^a^	P FDR adjust^a,b^
Epithelial tissue area						
CD3^+^CD4^+^ naive	1235	605 (49)	Tertiles	1.65 (1.22, 2.22)	0.001	**0.002**
CD3^+^CD4^+^ memory	1235	288 (23)	Quartiles	1.28 (0.96, 1.71)	0.093	0.119
CD3^+^CD4^+^ regulatory	1235	579 (47)	Tertiles	2.03 (1.51, 2.74)	3.5E-06	**1.3E-05**
CD3^+^CD8^+^ naive	1235	380 (31)	Tertiles	3.49 (2.57, 4.75)	1.2E-15	**2.1E-14**
CD3^+^CD8^+^ memory	1235	271 (22)	Quartiles	2.82 (2.10, 3.78)	4.4E-12	**2.6E-11**
CD3^+^CD8^+^ regulatory	1235	355 (29)	Tertiles	3.04 (2.24, 4.13)	1.2E-12	**1.1E-11**
CD3^+^CD4^-^CD8^-^ naive	1235	1187 (96)	Binary	2.41 (1.16, 4.89)	0.016	**0.024**
CD3^+^CD4^-^CD8^-^ memory	1235	1020 (83)	Binary	2.49 (1.68, 3.7)	5.4E-06	**1.6E-05**
CD3^-^ immune cells	1235	5 (<1)	Quartiles	2.10 (1.58, 2.79)	3.0E-07	**1.3E-06**
Stromal tissue area						
CD3^+^CD4^+^ naive	1233	206 (17)	Tertiles	1.14 (0.85, 1.53)	0.398	0.447
CD3^+^CD4^+^ memory	1233	23 (2)	Quartiles	0.68 (0.51, 0.91)	0.009	**0.015**
CD3^+^CD4^+^ regulatory	1233	182 (15)	Tertiles	1.43 (1.06, 1.93)	0.019	**0.027**
CD3^+^CD8^+^ naive	1233	198 (16)	Tertiles	1.72 (1.28, 2.32)	3.6E-04	**6.5E-04**
CD3^+^CD8^+^ memory	1233	77 (6)	Quartiles	1.90 (1.42, 2.54)	1.7E-05	**4.5E-05**
CD3^+^CD8^+^ regulatory	1233	209 (17)	Tertiles	1.78 (1.32, 2.4)	1.5E-04	**3.4E-04**
CD3^+^CD4^-^CD8^-^ naive	1233	976 (79)	Binary	1.28 (0.87, 1.86)	0.204	0.245
CD3^+^CD4^-^CD8^-^ memory	1233	586 (47)	Binary	1.14 (0.82, 1.6)	0.443	0.469
CD3^-^ immune cells	1233	0 (0)	Quartiles	0.93 (0.7, 1.23)	0.613	0.613

a OR (95% CI) and P values obtained from ordinal logistic regression when 3 or more quantiles were used, binary logistic regression when only two categories are used. Categories were assigned based on the larger percentage of zeros across both tissue types, where less than 25% zeros were categorized as quartiles, greater than 25% and less than 60% zeros were categorized to tertiles, and greater than 60% zeros were categorized as binary. Adjusted for age, sex, study batch (Harvard/OFCCR), and cancer site (proximal, distal, rectal, missing).

b P adjust = FDR adjusted for 18 tests: all subsets, epithelial and stromal as separate tests.

MSI, microsatellite instability; MSS, microsatellite stable; OR, odds ratio; CI, confidence interval.P FDR adjust < 0.05 are bolded.

**Table 3:** Association between microsatellite instability status and T cell subset densities, stratified by proliferation (MKI67) status.

Outcome	N	Proliferating Subsets					Non-Proliferating Subsets				
		Density Zeros N (%)	Categorization	OR per increasing quantile (95% CI) for MSI-high compared to MSI-low/MSS^a^	P^a^	P FDR adjust ^a,b^	Density Zeros N (%)	Categorization	OR per increasing quantile (95% CI) for MSI-high compared to MSI-low/MSS^a^	P^a^	P FDR adjust^a,b^
Epithelial tissue area											
CD3^+^CD4^+^ naive	1235	1054 (85)	Binary	1.76 (1.17, 2.65)	0.006	**0.012**	628 (51)	Binary	1.73 (1.25, 2.41)	0.001	**0.002**
CD3^+^CD4^+^ memory	1235	980 (79)	Binary	1.77 (1.23, 2.54)	0.002	**0.005**	295 (24)	Binary	1.03 (0.7, 1.53)	0.876	0.901
CD3^+^CD4^+^ regulatory	1235	1069 (86)	Binary	1.82 (1.18, 2.77)	0.006	**0.011**	593 (48)	Binary	2.03 (1.46, 2.82)	2.4E-05	**8.6E-05**
CD3^+^CD8^+^ naive	1235	718 (58)	Tertiles	3.27 (2.38, 4.5)	2.3E-13	**4.0E-12**	421 (34)	Tertiles	3.23 (2.39, 4.36)	2.2E-14	**8.0E-13**
CD3^+^CD8^+^ memory	1235	793 (64)	Binary	2.28 (1.65, 3.16)	5.3E-07	**3.8E-06**	296 (24)	Binary	3.04 (1.94, 4.94)	2.7E-06	**1.2E-05**
CD3^+^CD8^+^ regulatory	1235	710 (57)	Tertiles	2.75 (2, 3.76)	3.2E-10	**2.9E-09**	403 (33)	Tertiles	2.87 (2.11, 3.89)	1.2E-11	**1.4E-10**
CD3^+^CD4^-^CD8^-^ naive	1235	1230 (99.5)	Binary	1.11 (0.05, 11.18)	0.937	0.937	1191 (96)	Binary	2.73 (1.3, 5.66)	0.007	**0.012**
CD3^+^CD4^-^CD8^-^ memory	1235	1221 (99)	Binary	3.41 (1.05, 11.44)	0.041	0.059	1025 (83)	Binary	2.61 (1.75, 3.88)	2.1E-06	**1.2E-05**
CD3^-^ immune cells	1235	314 (25)	Tertiles	1.73 (1.29, 2.32)	2.7E-04	**7.3E-04**	8 (1)	Tertiles	2.04 (1.52, 2.73)	2.3E-06	**1.2E-05**
Stromal tissue area											
CD3^+^CD4^+^ naive	1233	709 (57)	Binary	1.46 (1.04, 2.06)	0.027	**0.043**	214 (17)	Binary	1.12 (0.73, 1.76)	0.605	0.729
CD3^+^CD4^+^ memory	1233	501 (41)	Binary	0.97 (0.71, 1.35)	0.875	0.901	27 (2)	Binary	0.18 (0.07, 0.43)	1.7E-04	**5.6E-04**
CD3^+^CD4^+^ regulatory	1233	707 (57)	Binary	1.09 (0.79, 1.5)	0.608	0.729	193 (16)	Binary	1.11 (0.72, 1.74)	0.640	0.743
CD3^+^CD8^+^ naive	1233	643 (52)	Tertiles	1.77 (1.3, 2.4)	3.0E-04	**7.3E-04**	214 (17)	Tertiles	1.73 (1.29, 2.34)	2.9E-04	**7.3E-04**
CD3^+^CD8^+^ memory	1233	550 (44)	Binary	1.64 (1.18, 2.28)	0.003	**0.006**	87 (7)	Binary	1.1 (0.61, 2.08)	0.754	0.848
CD3^+^CD8^+^ regulatory	1233	670 (54)	Tertiles	1.96 (1.44, 2.67)	1.8E-05	**7.3E-05**	227 (18)	Tertiles	1.76 (1.31, 2.37)	2.1E-04	**6.2E-04**
CD3^+^CD4^-^CD8^-^ naive	1233	1204 (97)	Binary	3.17 (1.26, 7.78)	0.012	**0.020**	986 (80)	Binary	1.24 (0.84, 1.81)	0.282	0.391
CD3^+^CD4^-^CD8^-^ memory	1233	1172 (95)	Binary	1.4 (0.71, 2.65)	0.314	0.418	594 (48)	Binary	1.17 (0.83, 1.63)	0.371	0.477
CD3^-^ immune cells	1233	71 (6)	Tertiles	1.39 (1.04, 1.88)	0.028	**0.043**	1 (<1)	Tertiles	0.96 (0.72, 1.29)	0.798	0.870

a OR (95% CI) and P values obtained from ordinal logistic regression when 3 or more quantiles were used, binary logistic regression when only two categories are used. Categories were assigned based on the larger percentage of zeros across all categories, where less than 25% zeros were categorized as quartiles, greater than 25% and less than 60% zeros were categorized to tertiles, and greater than 60% zeros were categorized as binary. Adjusted for age, sex, study batch (Harvard/OFCCR), and cancer site (proximal, distal, rectal, missing).

b P adjust = FDR adjusted for 36 tests: all subsets, epithelial and stromal as separate tests, proliferating and non-proliferating.

MSI, microsatellite instability; MSS, microsatellite stable; OR, odds ratio; CI, confidence interval.P FDR adjust < 0.05 are bolded.

## Results

### Participant cohort and experimental approach

The mean age at diagnosis (standard deviation) of participants was 67 (9) years (**Supplementary Table 2**). Proportions of both sexes were roughly equal, where 54% of our sample were female. A majority of included participants had tumors located in the colon (77%), and were diagnosed at stages II or III (60%). In total, 218 out of 1,236 tumors were classified as MSI-high (18%), and among those with targeted sequencing data, 107 (17%) were hypermutated. Compared to MSI-low/MSS cases, MSI-high cases were more likely to be female, belong to the NHS study, have tumors located in the proximal colon, and be diagnosed at stage II or III (**Supplementary Table 2**).

In this study we identified 2,627,801 total immune cells, including 572,163 CD3^+^ T cells. One participant did not have epithelial tissue regions and three participants did not have any stromal tissue regions present in their TMA core, resulting in 1,235 participants for tests of epithelial tissue area and 1,233 for tests of stromal tissue area. A schematic outlining our T cell populations identified, example epithelial tissue areas and stromal tissue areas, and example cell segmentation and multiplex images are presented in **Supplementary Figure 1**. T cell subsets were overdispersed and right-skewed (**Supplementary Figure 2**). Stromal tissue regions generally had higher cell densities than epithelial regions for each T cell subset. The percentage of zeros for each T cell subset was generally higher in epithelial than stromal tissue regions (**Table 2**). Double-negative (CD3^+^CD4^-^CD8^-^) naïve and memory T cells in epithelial areas were the rarest subsets, resulting in the most zeros (96% and 83% zeros, respectively). CD3^+^CD4^+^ and CD3^+^CD8^+^ T cells had more modest zero percentages, ranging from 2% to 49%.

### T cell density differences by MSI status

**Figure 1:**
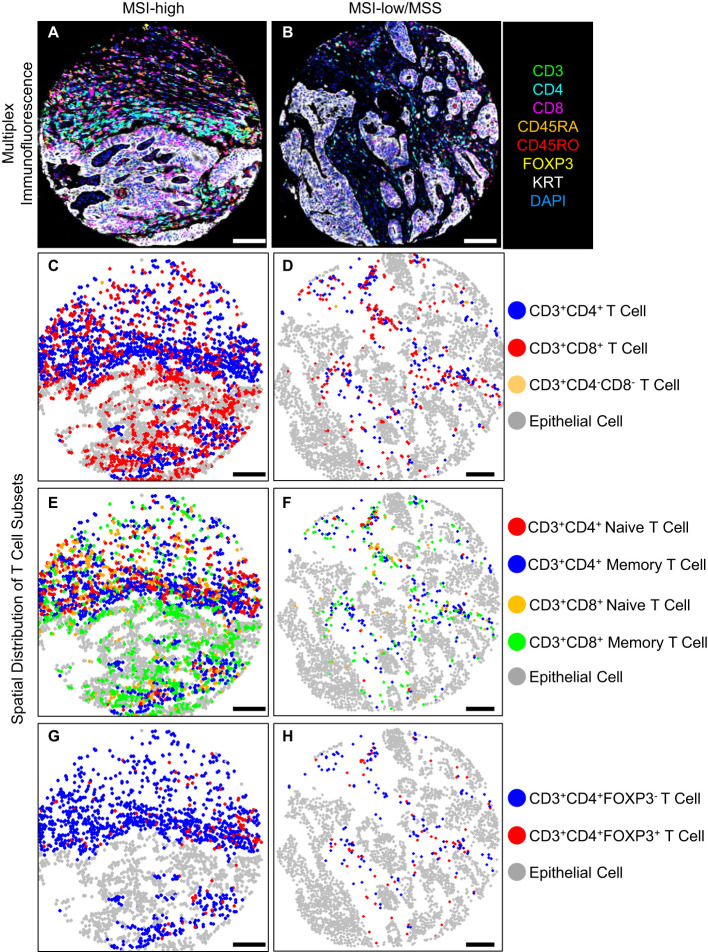
T cell density levels in an MSI-high tumor compared to an MSI-low/MSS tumor, **(A, B)** multiplex immunofluorescence, **(C-H)** spatial distribution of T cell subsets, scale bar 100 (μm).

**Figure 2:**
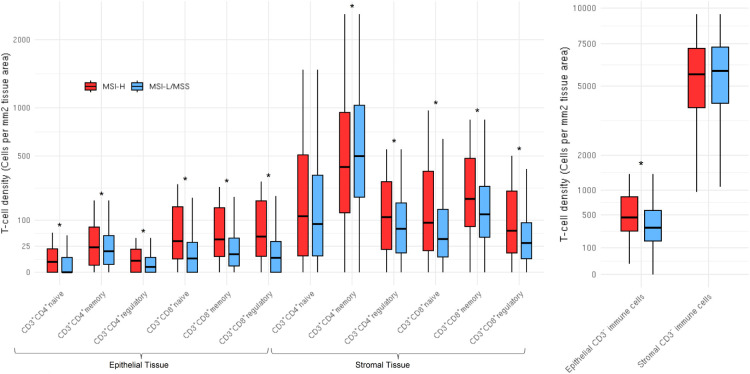
Distributions of T cell subsets and CD3^-^ immune cells stratified by microsatellite instability status in both epithelial (N=1,235) and stromal (N=1,233) tissue areas. CD3^+^CD4^-^CD8^-^ (double negatives) distributions not shown due to high percentage of zeros. Distributions are winsorized to their 95th percentile for visualization purposes. The box denotes the interquartile range with a line for median value, and the length of the vertical line represents 1.5 times the smallest value below 25th and 1.5 times the largest value above the 75th percentiles. Stars represent subsets that were significantly different; all subsets were significantly (pFDR < 0.05) different by MSI status, except stromal tissue area CD3^+^CD4^+^ naive T cells and stromal tissue area CD3^-^ immune cells.

Representative multiplex immunofluorescence images of an MSI-high tumor and MSI-low/MSS tumor are shown in **Figure 1**. MSI-high tumors had significantly (Wilcoxon pFDR adjusted < 0.05) higher densities of all CD3^+^CD4^+^ and CD3^+^CD8^+^ T cells in epithelial tissue areas, as well as higher densities of CD3^+^CD4^+^ regulatory T cells and all CD3^+^CD8^+^ T cell subsets in stromal areas compared to MSI-low/MSS tumors (**Figure 2**). MSI-high tumors had significantly lower densities of CD3^+^CD4^+^ memory T cells in stromal tissue areas compared to MSI-low/MSS.

In ordinal logistic models adjusted for age, sex, study batch, and cancer site, MSI-high status was associated with greater odds of higher density quantile for CD3^+^CD4^+^ naive and regulatory subsets, all CD3^+^CD8^+^ subsets, double negative T cells, and CD3^-^ non-tumor cells in epithelial tissue areas (**Table 2**). MSI-high status was also associated with greater odds of higher quantile for CD3^+^CD4^+^ regulatory T cells and all CD3^+^CD8^+^ subsets in stromal tissue areas, but with lower quantile for stromal area CD3^+^CD4^+^ memory T cells [OR per increasing quantile (95% CI) = 0.68 (0.51, 0.91), **Table 2**]. The strongest associations were for CD3^+^CD8^+^ T cells in epithelial areas, where those with MSI-high tumors had around 3 times the odds of greater quantile of epithelial tissue area CD3^+^CD8^+^ T cell densities compared to MSI-low/MSS tumors [OR per increasing quantile (95% CI) for CD3^+^CD8^+^ naive, memory, and regulatory subsets = 3.49 (2.57, 4.75); 2.82 (2.10, 3.78); 3.04 (2.24, 4.13), respectively].

Associations between MSI status and T cell subsets were generally similar across strata defined by proliferation status, with few exceptions (**Table 3**). In particular, the association of MSI status with CD3^+^CD4^+^ memory T cell densities in epithelial and stromal tissue areas differed by proliferation status. In epithelial tissue areas, MSI-high status was more strongly associated with higher levels of proliferating CD3^+^CD4^+^ memory T cells than with non-proliferating CD3^+^CD4^+^ memory T cells. In stromal tissue areas, MSI status was more strongly associated with lower levels of non-proliferating CD3^+^CD4^+^ memory T cells than proliferating CD3^+^CD4^+^ memory T cells. Double negative naive T cells were also differentially associated with MSI status by both tissue area and proliferation status.

### T cell density differences by hypermutation status

In the subset of participants who had hypermutation status available (N=639), 108 individuals were MSI-high, 107 were hypermutated, and 99 were both MSI-high and hypermutated. Among the 639 samples with available data, only 17 were discordant for their MSI-high and hypermutation statuses (**Supplementary Table 2**). Associations between hypermutation status and T cell subsets were largely consistent with associations found for MSI status (**Table 4**). Notable exceptions were epithelial area CD3^+^CD4^+^ memory and double negative naive T cells: epithelial area CD3^+^CD4^+^ memory cells were associated with hypermutation status but not with MSI status [hypermutation status OR (95% CI) = 1.68 (1.11, 2.53), MSI OR = 1.28 (0.96, 1.71)], while epithelial double negative naive T cells were associated with MSI but not hypermutation status [hypermutation status OR (95% CI) = 1.54 (0.46, 4.48), MSI OR = 2.41 (1.16, 4.89), **Tables 2** and **4**]. When examining total tumor mutational burden (indels and single nucleotide variants) continuously, we observed strong correlations with overall CD3^+^CD8^+^ T cells in both epithelial and stromal areas but not with epithelial or stromal area overall CD3^+^CD4^+^ T cells (**Supplementary Figure 3**).

**Table 4:** Association between hypermutation status and T cell subset densities.

Outcome	N	Density Zeros N (%)	Categorization	OR per increasing quantile (95% CI) for hypermutated compared to non-hypermutated tumors^a^	P^a^	P FDR adjust^a,b^
Epithelial tissue area						
CD3^+^CD4^+^ naive	639	314 (49)	Tertiles	1.69 (1.11, 2.58)	0.015	**0.024**
CD3^+^CD4^+^ memory	639	121 (19)	Quartiles	1.68 (1.11, 2.53)	0.014	**0.024**
CD3^+^CD4^+^ regulatory	639	279 (44)	Tertiles	1.8 (1.18, 2.76)	0.006	**0.013**
CD3^+^CD8^+^ naive	639	196 (31)	Tertiles	3.39 (2.20, 5.22)	3.2E-08	**5.7E-07**
CD3^+^CD8^+^ memory	639	129 (20)	Quartiles	3.00 (1.98, 4.55)	2.2E-07	**1.3E-06**
CD3^+^CD8^+^ regulatory	639	173 (27)	Tertiles	3.2 (2.08, 4.93)	1.4E-07	**1.2E-06**
CD3^+^CD4^-^CD8^-^ naive	639	615 (96)	Binary	1.54 (0.46, 4.48)	0.448	0.538
CD3^+^CD4^-^CD8^-^ memory	639	519 (81)	Binary	3.06 (1.81, 5.19)	3.1E-05	**1.4E-04**
CD3^-^ immune cells	639	3 (0)	Quartiles	2.33 (1.55, 3.49)	4.2E-05	**1.5E-04**
Stromal tissue area						
CD3^+^CD4^+^ naive	637	117 (18)	Tertiles	0.95 (0.63, 1.45)	0.827	0.827
CD3^+^CD4^+^ memory	637	10 (2)	Quartiles	0.67 (0.45, 1.01)	0.055	0.082
CD3^+^CD4^+^ regulatory	637	84 (13)	Tertiles	1.30 (0.86, 1.99)	0.215	0.290
CD3^+^CD8^+^ naive	637	95 (15)	Tertiles	1.88 (1.23, 2.86)	0.003	**0.007**
CD3^+^CD8^+^ memory	637	39 (6)	Quartiles	2.19 (1.44, 3.32)	2.2E-04	**6.7E-04**
CD3^+^CD8^+^ regulatory	637	101 (16)	Tertiles	2.07 (1.35, 3.17)	9.2E-04	**0.002**
CD3^+^CD4^-^CD8^-^ naive	637	501 (78)	Binary	1.12 (0.65, 1.90)	0.673	0.713
CD3^+^CD4^-^CD8^-^ memory	637	288 (45)	Binary	1.35 (0.84, 2.2)	0.225	0.29
CD3^-^ immune cells	637	0 (0)	Quartiles	0.92 (0.61, 1.37)	0.673	0.713

a OR (95% CI) and P values obtained from ordinal logistic regression when 3 or more quantiles were used, binary logistic regression when only two categories are used. Adjusted for age, sex, study batch (Harvard/OFCCR), and cancer site (proximal, distal, rectal, missing).

b P adjust = FDR adjusted for 18 tests: all subsets, epithelial and stromal as separate tests.

OR, odds ratio; CI, confidence interval.P FDR adjust < 0.05 are bolded.

## Discussion

In this large population-based study of T cell densities in CRC, MSI-high status was associated with higher density quantile for the majority of immune subsets in both epithelial and stromal tissue areas. The notable exception was CD3^+^CD4^+^ memory T cells in stromal areas, where MSI-high status was significantly associated with lower quantile of that subset compared to MSI-low/MSS tumors. CD3^+^CD8^+^ T cells in epithelial areas were particularly strongly associated with MSI-high status. Associations were largely consistent stratifying by proliferation status and examining hypermutation status as the main exposure of interest.

Our results are consistent with previous work, where MSI status is well-known to be associated with overall T cell response in CRC (10–13). Although few studies have examined associations with T cell subsets, at least one prior study noted a specific positive association of CD45RO^+^ memory T cells with MSI-high status (14). FOXP3^+^ regulatory T cells have also been found to have higher intraepithelial infiltration in MSI-high tumors compared to in MSS tumors (31). MSI status is a critical variable to examine in CRC, as it reflects a somatic deficiency in DNA mismatch repair resulting in high tumor mutation burden and, therefore, high neoantigen presentation. In particular, MSI-high tumors, with accumulated somatic mutations, may modulate immune response through upregulated expression of immune checkpoints (26). As such, MSI status is also known to be strongly associated with treatment response, particularly treatment with immune checkpoint inhibitors (4–6), and survival (32–34). Interestingly, a recent meta-analysis of 13,029 patients combining both MSI status and tumor infiltrating lymphocytes (TILs) found that better survival was only observed among CRC patients that were TILs-high, regardless of MSI status- although patients with MSI-high TILs-low did still have better survival than those MSS TILs-low, particularly for CRC-specific survival (35). This study supports efforts to include both T cell densities and MSI status in clinical decision making.

Our study examines the association between MSI status and T cell specific subset densities within both epithelial and stromal tissue. We prioritized identifying non-overlapping subsets of naive, memory, and regulatory both CD3^+^CD4^+^ and CD3^+^CD8^+^ subsets of T cells due to the different roles these cell types play in the tumor immune microenvironment. For example, CD3^+^CD4^+^ T cells support the expansion and differentiation of CD3^+^CD8^+^ T cells, as well as conducting surveillance, regulating tissue homeostasis, and restricting adaptive immune response (36). In contrast, CD3^+^CD8^+^ T cells kill neoplastic cells and pathogens to drive anti-cancer immune response (37). T cells are functionally considered naive until they encounter their specific antigen in peripheral tissues, after which they differentiate into either effector or memory T cells (38). Naïve and memory T cells have been found to have different chromatin accessibility and transcription factor expression (38). Regulatory T cells, primarily CD3^+^CD4^+^FOXP3^+^ T cells, are involved in immune homeostasis and auto-immunity prevention (39). In addition to differential roles of different subsets, the location of T cells is also critical and influences function (40). For example, only T cells within tumor epithelial areas, versus stromal areas, can eliminate tumor cells through direct cell-cell contact (40–43). The heterogeneity of the tumor immune microenvironment, both through cell function and location, indicate the importance of examining associations critically between immune cell populations and predictor or outcomes of interest. For example, in our analysis we found that MSI-high tumors had 2 times the odds of greater quantile of epithelial tissue area CD3^+^CD4^+^ regulatory T cell densities compared to MSI-low/MSS tumors, which may have been obscured if CD3^+^CD4^+^ T cell densities were examined overall. Examining immune subsets in broad, generalized categories may obscure subset-specific associations that are informative of immune response biology.

Although the majority of T cell subsets were observed to be positively associated with MSI-high status in our analysis, we also found that MSI-high tumors had lower quantile densities of CD3^+^CD4+ memory T cells in stromal areas. Memory CD3^+^CD4^+^ T cells are derived from a cell population that experienced a specific antigen but remained in the body after said antigen was no longer present, where they may be protective against cancer but also be involved in auto-immunity, allergy, and chronic inflammation (36). Although the basis for our observed inverse finding between density of this subset and MSI-high status is not clear, this unexpected association may be influenced by other immune cells in the tumor microenvironment that were not profiled as a part of our study- for example, macrophages or dendritic cells. Further research is needed on how MSI-high status impacts memory CD3^+^CD4^+^ T cells in stromal tissue regions.

The differential responsiveness of MSI-high compared to MSI-low/MSS CRCs to immune checkpoint blockade treatment provides strong evidence that MSI status is a clinically relevant tumor characteristic (44). While MSI-high tumors clearly had statistically significantly higher T cell densities for many subsets in our study, it is important to note that the absolute difference between MSI-high and MSI-low/MSS is relatively modest. MSI-low/MSS tumors still had reasonable densities of many T cell populations that were not orders of magnitude different from those observed in MSI-high tumors. Moreover, the previously mentioned meta-analysis combining both TILs and MSI status in relation to survival found that the MSS TILs-high subtype had CRC-specific survival hazard ratios that were very comparable to those of MSI-high TILs-high (HR=0.55, 0.47-0.64 and HR=0.53, 0.43-0.66, respectively) (35), suggesting that MSS tumors with high levels of T cell densities may behave more similarly to MSI-high tumors. This finding may provide motivation for future treatments to better exploit the T cell response present in MSI-low/MSS CRC tumors.

Our study has notable strengths. This is the first study, to our knowledge, to examine the association of MSI and hypermutation status with specific non-overlapping T cell subsets using a mIF assay, where we have the ability to determine non-overlapping specific naive, memory, and regulatory subsets of cytotoxic and helper T cells in both epithelial and stromal tissue areas, as well as double negative T cells and CD3^-^ immune cells. Our integrated CRC tumor databases (15, 16) enabled us to link MSI and hypermutation status with the features of the tumor microenvironment while accounting for demographic and epidemiologic variables. We also acknowledge several limitations in this study. The vast majority of our sample was non-Hispanic White individuals, necessitating future studies in other populations. Additionally, while our study is notable in its inclusion of specific T cell density subsets that include differentiation by naive, memory, or regulatory subtype, as well as proliferation status- our study only includes information on T cell densities (i.e., cell count per mm^2^) and broad tissue location (i.e., tumor intraepithelial vs. stromal regions), rather than additionally including information on spatial biology of the tumor. Spatial organization of the tumor immune microenvironment has been shown to be independently prognostic of CRC outcomes (45–49). In future work, detailed data on the spatial biology from both TMAs as well as whole slides will allow us to more comprehensively address this question of spatial organization of the tumor immune microenvironment in relation to survival and tumor characteristics. Previous work has suggested that assessment of tumor structures is more powerfully addressed through whole-slide imaging, rather than TMAs (50). We acknowledge the limitation of our study in using TMAs to examine the tumor microenvironment, however, TMA sections contain relevant information and vastly improve researchers ability to conduct studies of immune profiling in large scale population studies, where multiplex imaging analysis of whole slide images is cost prohibitive. Additionally, the use of TMAs expanding our sample size allows us to examine different subset breakdowns, such as quartiles of T cell densities among MSI-high tumors, which we may not be able to examine otherwise with smaller numbers due to low statistical power. Furthermore, in future work we aim to compare whole slide imaging with our TMA results to compare our findings utilizing different methods. In future work we also aim to examine mechanisms of immune cell recruitment and immune checkpoint molecules as these are important pathways that are likely at play in the relationship between MSI status and T cell densities.

In conclusion, MSI-high and hypermutation status were strongly associated with different T cell and immune subsets in both epithelial and stromal tissue areas, where the strongest associations were present for CD3^+^CD8^+^ T cells in epithelial tissue areas. Our large-scale investigation indicates potential mechanisms by which MSI-high status confers better survival and supports ongoing and future efforts to identify patients who may be more likely to respond to immunotherapy treatments.

## Data Availability

The raw data supporting the conclusions of this article will be made available by the authors, without undue reservation.

## Supplementary material

Supplementary Table 1 Antibodies and Staining Conditions for Multiplex Immunofluorescence Histological Analysis.

Supplementary Table 2 Participant characteristics overall and by MSI status. *****MSI = microsatellite instability high (MSI-high), low (MSI-low), or microsatellite stable (MSS). P values were calculated using t test for mean differences & chi square for frequencies.

Supplementary Figure 1
**(A)** Profiling of the multiplexed immunofluorescence panel, utilizing antibodies targeting CD3, CD4, CD8, CD45RA, CD45RO, FOXP3, KRT, and DAPI. **(B-E)**. Multiplex immunofluorescence images **(B, C)** were analyzed to classify both tissue and cellular categories. Tissue segmentation **(D)** was performed to delineate epithelial (red), stromal (green), and other (blue) regions. Cell segmentation **(E)** was then sequentially applied to classify individual cells. Scale bar: 100 (μm). **(F)** Identification of distinct T-cell subsets via the co-expression of T-cell markers (membrane CD3, membrane CD4, membrane CD8, membrane CD45RA, membrane CD45RO, nucleus FOXP3), epithelial marker (cytoplasm KRT), and DNA marker (nucleus DAPI) at single-cell resolution.

Supplementary Figure 2 Boxplot and beeswarm plot distributions of **(A)** T cell subsets and **(B)** CD3^-^ immune cells stratified by epithelial (N=1,235) and stromal (N=1,233) tissue area. CD3^+^CD4^-^CD8^-^ (double negatives) distributions not shown due to high percentage of zeros. The box denotes the inter-quartile range with a line for median value, and the length of the vertical line represents 1.5 times the smallest value below 25th and 1.5 times the largest value above the 75th percentiles.

Supplementary Figure 3 Scatter plots and correlation of tumor mutational burden and T cell densities. Footnote: N = 639 in epithelial areas, N = 637 in stromal areas.
