# Supplementary material for: Association between somatic microsatellite instability, hypermutation status, and specific T cell subsets in colorectal cancer tumors
Source: Front Immunol. 2024 Dec 23;15:1505896. doi: 10.3389/fimmu.2024.1505896 (PMC11701007; doi:10.3389/fimmu.2024.1505896)
Supplement: Supplementary Table 1 — Antibodies and Staining Conditions for Multiplex Immunofluorescence Histological Analysis. [file DataSheet1.pdf]

Supplemental Tables & Figures: Association between somatic microsatellite instability, hypermutation status, and specific T cell subsets in colorectal cancer tumors

Authors:
Claire E. Thomas, Yasutoshi Takashima, Evertine Wesselink, Tomotaka Ugai, Robert S. Steinfelder, Daniel D. Buchanan, Conghui Qu, Li Hsu, Andressa Dias Costa, Steven Gallinger, Robert C. Grant, Jeroen R. Huyghe, Sushma S. Thomas, Shuji Ogino, Amanda I. Phipps, Jonathan A. Nowak, Ulrike Peters

Supplemental Table 1: Antibodies and Staining Conditions for Multiplex Immunofluorescence Histological Analysis

Staining Order	Targeted Antigen	Epitope Retrieval	Antibody Clone and Supplier	Species	Dilution	Opal Dye	Opal Dye Dilution
1	CD3	ER1	F7.2.38, Agilent Dako (Santa Clara, CA, USA)	Mouse Monoclonal	1:50	540	1:150
2	CD4	ER2	EP204, Cell Marque (Rocklin, CA, USA)	Rabbit Monoclonal	1:25	520	1:100
3	MKI67	ER1	SP6, Invitrogen (Waltham, MA, USA)	Rabbit Monoclonal	1:300	480	1:300
4	FOXP3	ER1	236A/E7, abcam (Waltham, MA, USA)	Mouse Monoclonal	1:100	570	1:400
5	CD8	ER1	C8/144B, Agilent Dako (Santa Clara, CA, USA)	Mouse Monoclonal	1:150	650	1:300
6	CD45RA	ER1	HI100, Biolegend (San Diego, CA, USA)	Mouse Monoclonal	1:1000	620	1:100
7	CD45RO	ER1	UCHL1, Agilent Dako (Santa Clara, CA, USA)	Mouse Monoclonal	1:50	690	1:300
8	KRT	ER1	C11, Cell Signaling (Beverly, MA, USA)<br>AE1/AE3, Agilent Dako (Santa Clara, CA, USA)	Mouse Monoclonal<br>Mouse Monoclonal	1:400<br>1:40	780<br>DIG	1:25<br>1:100

Supplemental Table 2: Participant characteristics overall and by MSI status

Characteristic	Overall, N = 1,236	MSI-high, N=218	MSI-low/MSS, N=1,018	P-value
Mean age at diagnosis ± standard deviation (years)	67 (9)	68 (9)	67 (9)	0.277
Sex, N (%)				
Female	662 (54)	149 (68)	513 (50)	2.0e-06
Male	574 (46)	69 (32)	505 (50)	
Study, N (%)				
HPFS	411 (33)	49 (22)	362 (36)	8.4e-04
NHS	498 (40)	105 (48)	393 (39)	
OFCCR	327 (26)	64 (29)	263 (26)	
Site, N (%)				
Proximal	577 (47)	183 (84)	394 (39)	<2.2e-16
Distal	373 (30)	22 (10)	351 (34)	
Rectal	270 (22)	8 (4)	262 (26)	
Missing	16 (1)	5 (2)	11 (1)	
Stage, N (%)				
Stage I, local	257 (21)	42 (19)	215 (21)	3.5e-05
Stage II or III, regional	743 (60)	158 (72)	585 (57)	
Stage IV, distant	158 (13)	12 (5)	146 (14)	
Missing	78 (6)	6 (3)	72 (7)	
MSI status*, N (%)				
MSI-high	218 (18)			
MSI-low/MSS	1018 (82)			
Hypermutation status (among subset with available data), N (%)	Total N in subset = 639	N=108	N=531	
Hypermutated	107 (17)	99 (92)	8 (2)	<2.2e-16
Non-hypermutated	532 (83)	9 (8)	523 (98)	

*****MSI = microsatellite instability high (MSI-high), low (MSI-low), or microsatellite stable (MSS).

P values were calculated using t test for mean differences & chi square for frequencies.

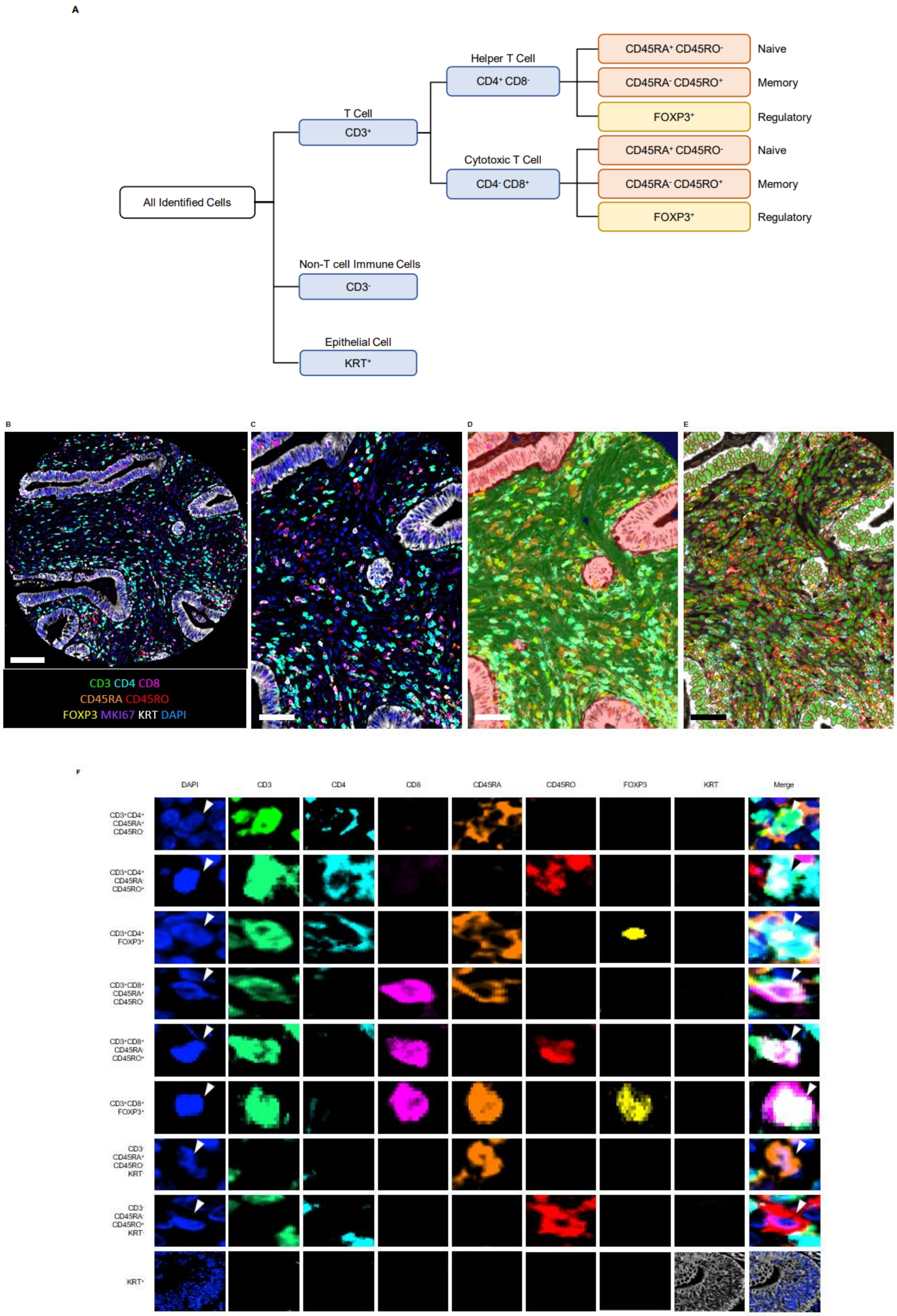

Supplemental Figure 1: (A). Profiling of the multiplexed immunofluorescence panel, utilizing antibodies targeting CD3, CD4, CD8, CD45RA, CD45RO, FOXP3, KRT, and DAPI. (B-E). Multiplex immunofluorescence images (B-C) were analyzed to classify both tissue and cellular categories. Tissue segmentation (D) was performed to delineate epithelial (red), stromal (green), and other (blue) regions. Cell segmentation (E) was then sequentially applied to classify individual cells. Scale bar: 100 (µm). (F) Identification of distinct T-cell subsets via the co-expression of T-cell markers (membrane CD3, membrane CD4, membrane CD8, membrane CD45RA, membrane CD45RO, nucleus FOXP3), epithelial marker (cytoplasm KRT), and DNA marker (nucleus DAPI) at single-cell resolution.

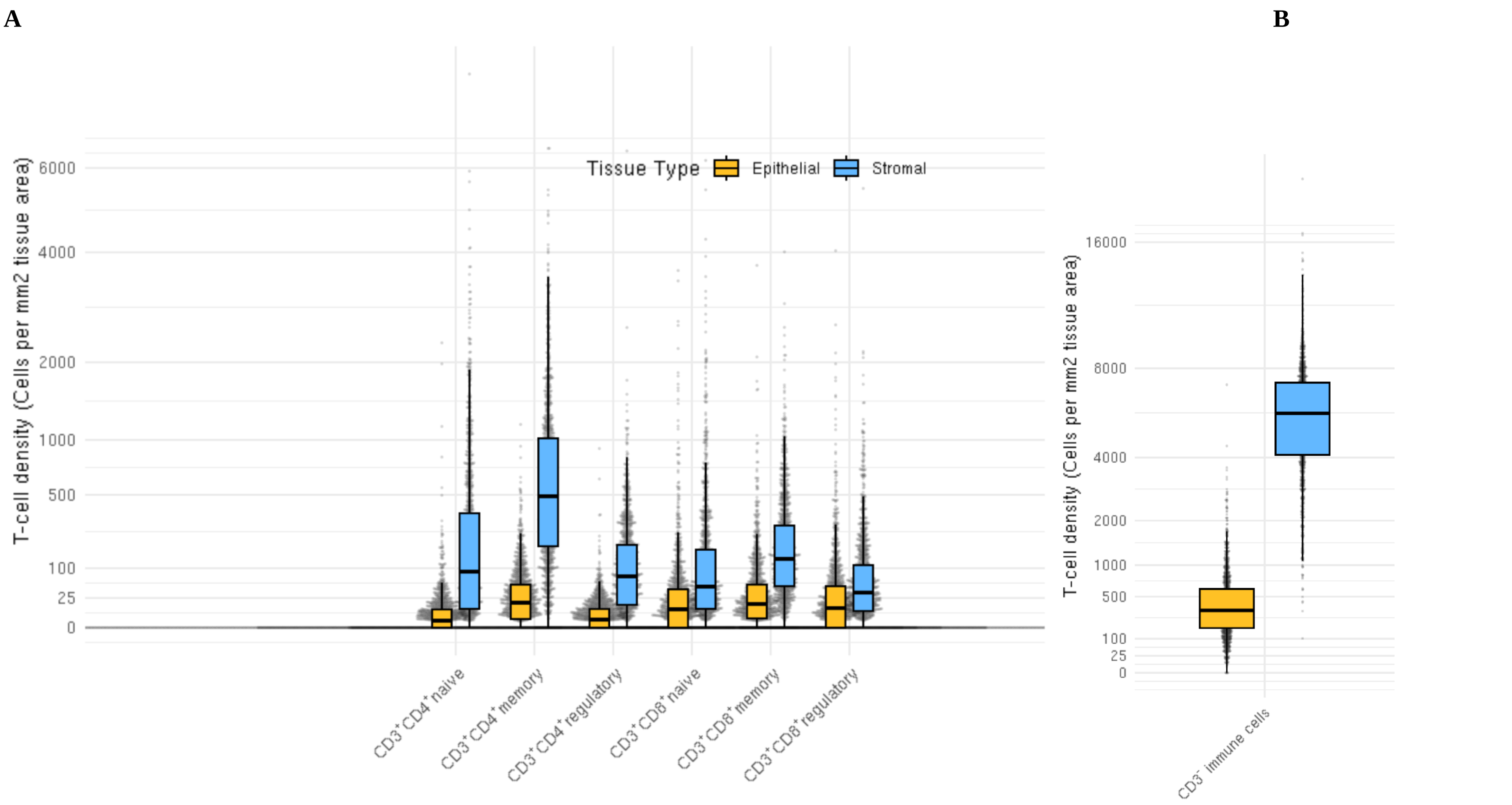

Supplemental Figure 2: Boxplot and beeswarm plot distributions of A) T cell subsets and B) $CD3^-$ non-tumor cells stratified by epithelial (N=1,235) and stromal (N=1,233) tissue area. $CD3^+CD4^-CD8^-$ (double negatives) distributions not shown due to high percentage of zeros. The box denotes the inter-quartile range with a line for median value, and the length of the vertical line represents 1.5 times the smallest value below 25th and 1.5 times the largest value above the 75th percentile

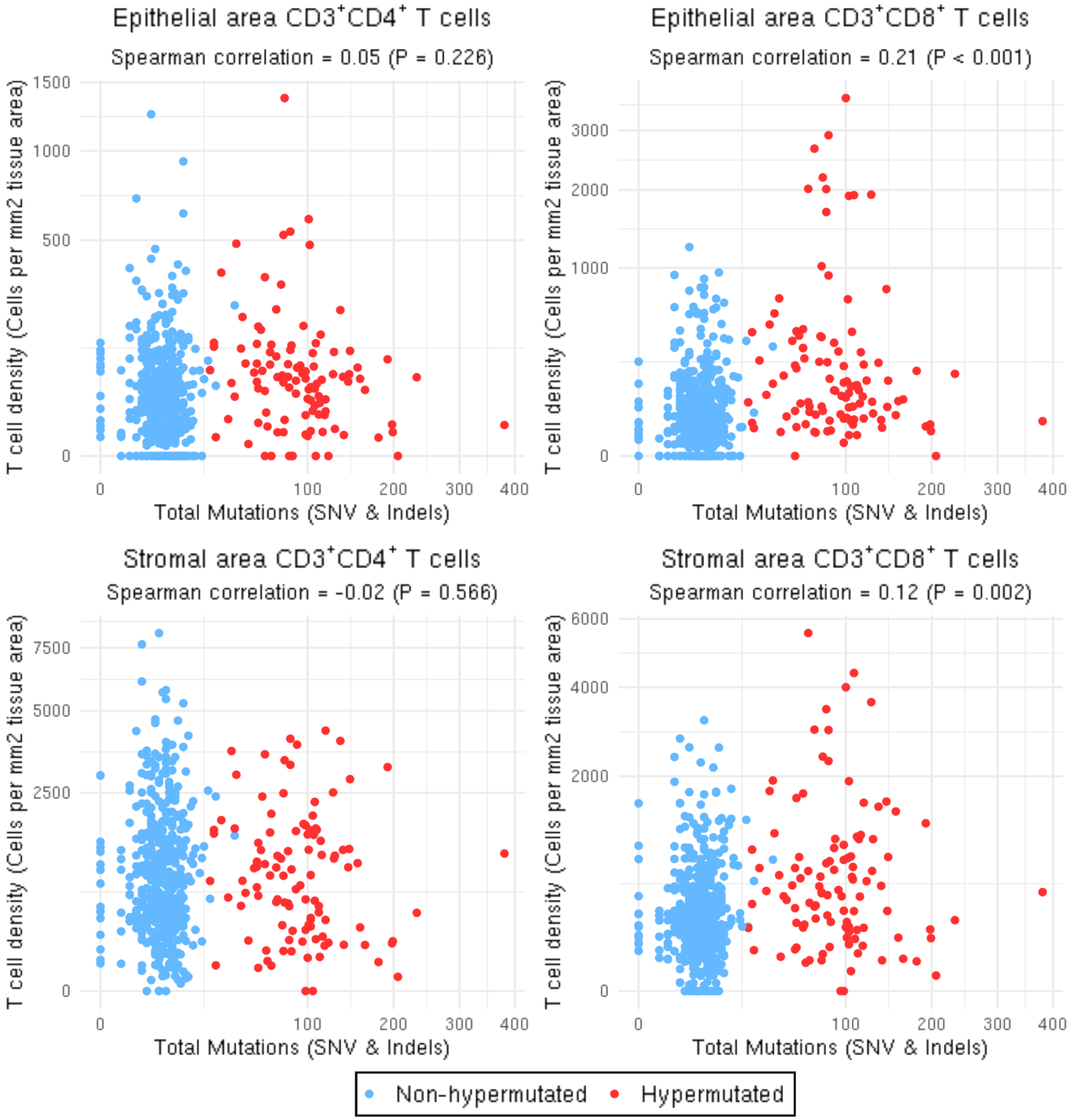

Supplemental Figure 3: Scatter plots and correlation of tumor mutational burden and T cell densities

Footnote: N = 639 in epithelial areas, N = 637 in stromal areas.
