# Supplementary figures and images for: Association between somatic microsatellite instability, hypermutation status, and specific T cell subsets in colorectal cancer tumors
Source: Front Immunol. 2024 Dec 23;15:1505896. doi: 10.3389/fimmu.2024.1505896 (PMC11701007; doi:10.3389/fimmu.2024.1505896)

Supplemental Figure I

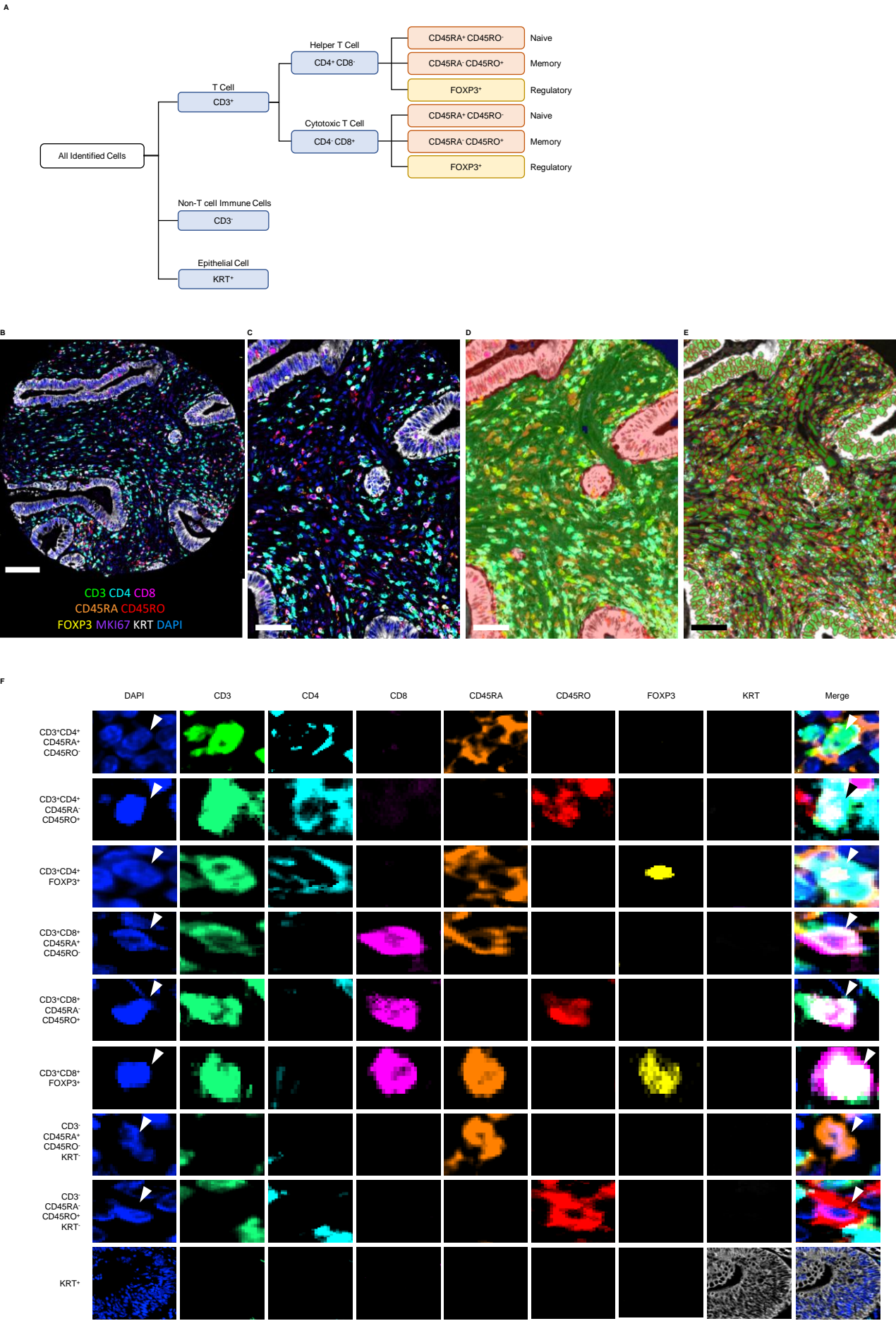

Supplement: Supplementary Figure 1 — (A) Profiling of the multiplexed immunofluorescence panel, utilizing antibodies targeting CD3, CD4, CD8, CD45RA, CD45RO, FOXP3, KRT, and DAPI. (B-E). Multiplex immunofluorescence images (B, C) were analyzed to classify both tissue and cellular categories. Tissue segmentation (D) was performed to delineate epithelial (red), stromal (green), and other (blue) regions. Cell segmentation (E) was then sequentially applied to classify individual cells. Scale bar: 100 (μm). (F) Identification of distinct T-cell subsets via the co-expression of T-cell markers (membrane CD3, membrane CD4, membrane CD8, membrane CD45RA, membrane CD45RO, nucleus FOXP3), epithelial marker (cytoplasm KRT), and DNA marker (nucleus DAPI) at single-cell resolution. [file Image1.pdf]

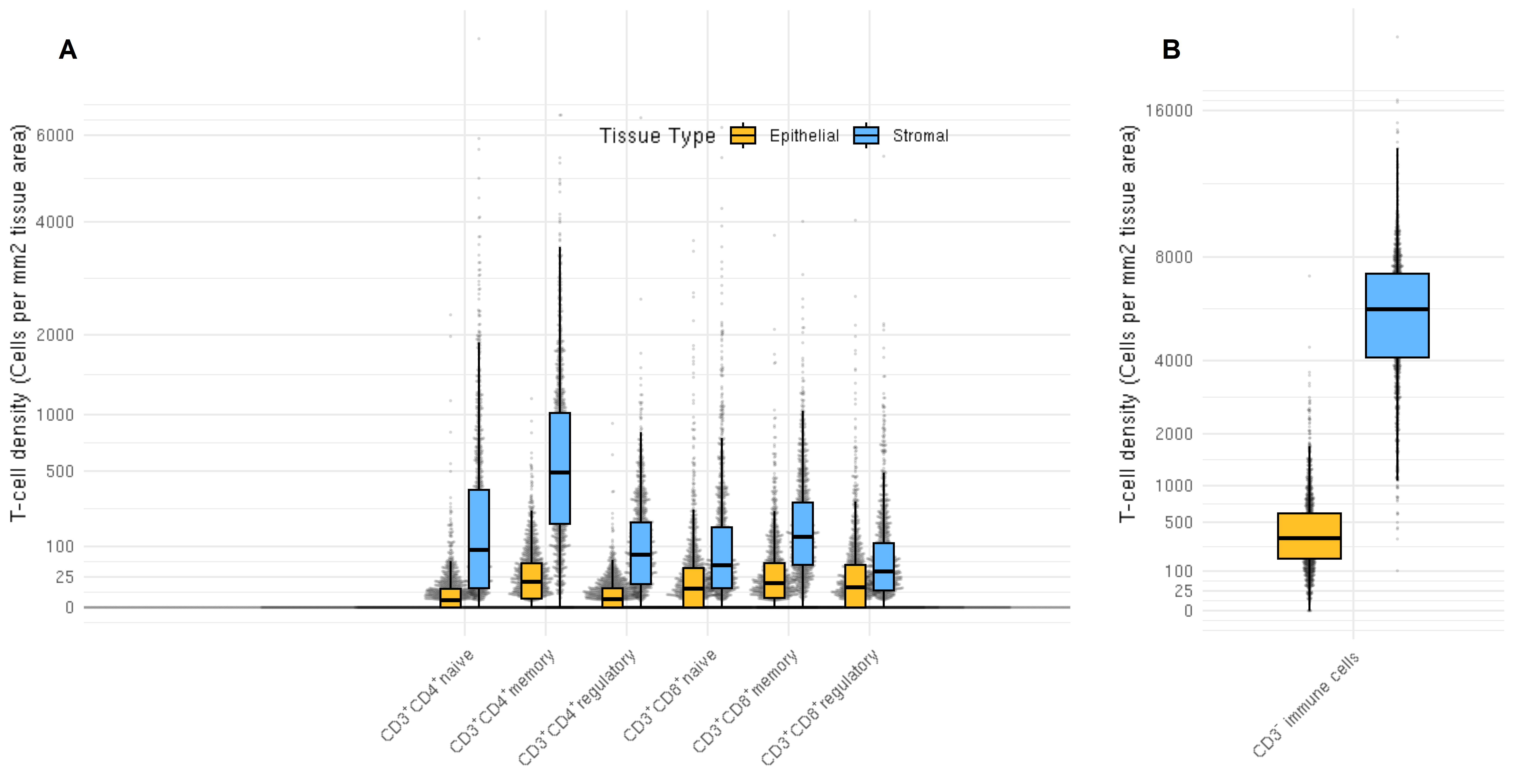

Supplement: Supplementary Figure 2 — Boxplot and beeswarm plot distributions of (A) T cell subsets and (B) CD3- immune cells stratified by epithelial (N=1,235) and stromal (N=1,233) tissue area. CD3+CD4-CD8- (double negatives) distributions not shown due to high percentage of zeros. The box denotes the inter-quartile range with a line for median value, and the length of the vertical line represents 1.5 times the smallest value below 25th and 1.5 times the largest value above the 75th percentiles. [file Image2.png]

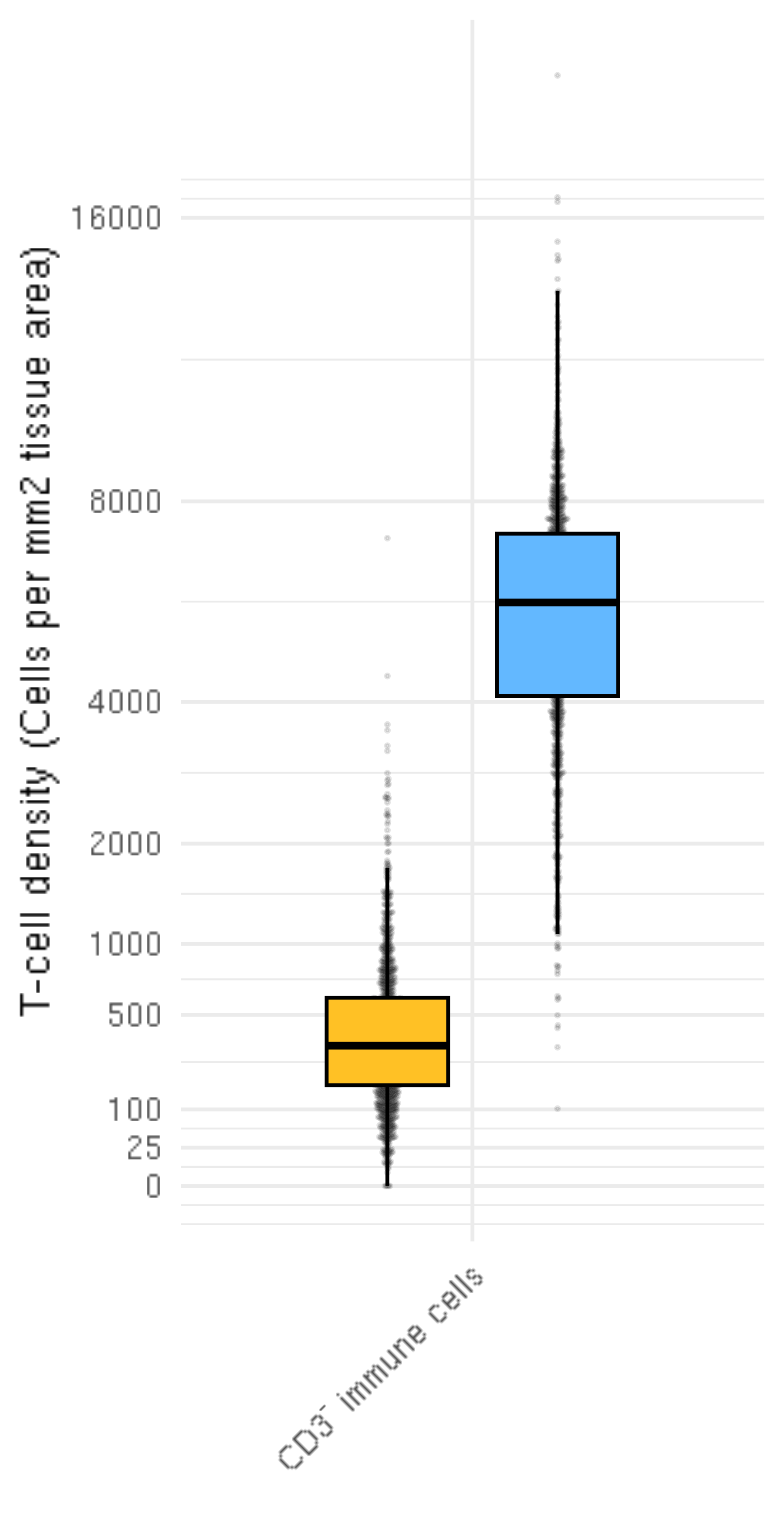

Supplement: Supplementary file 4 [file Image3.png]

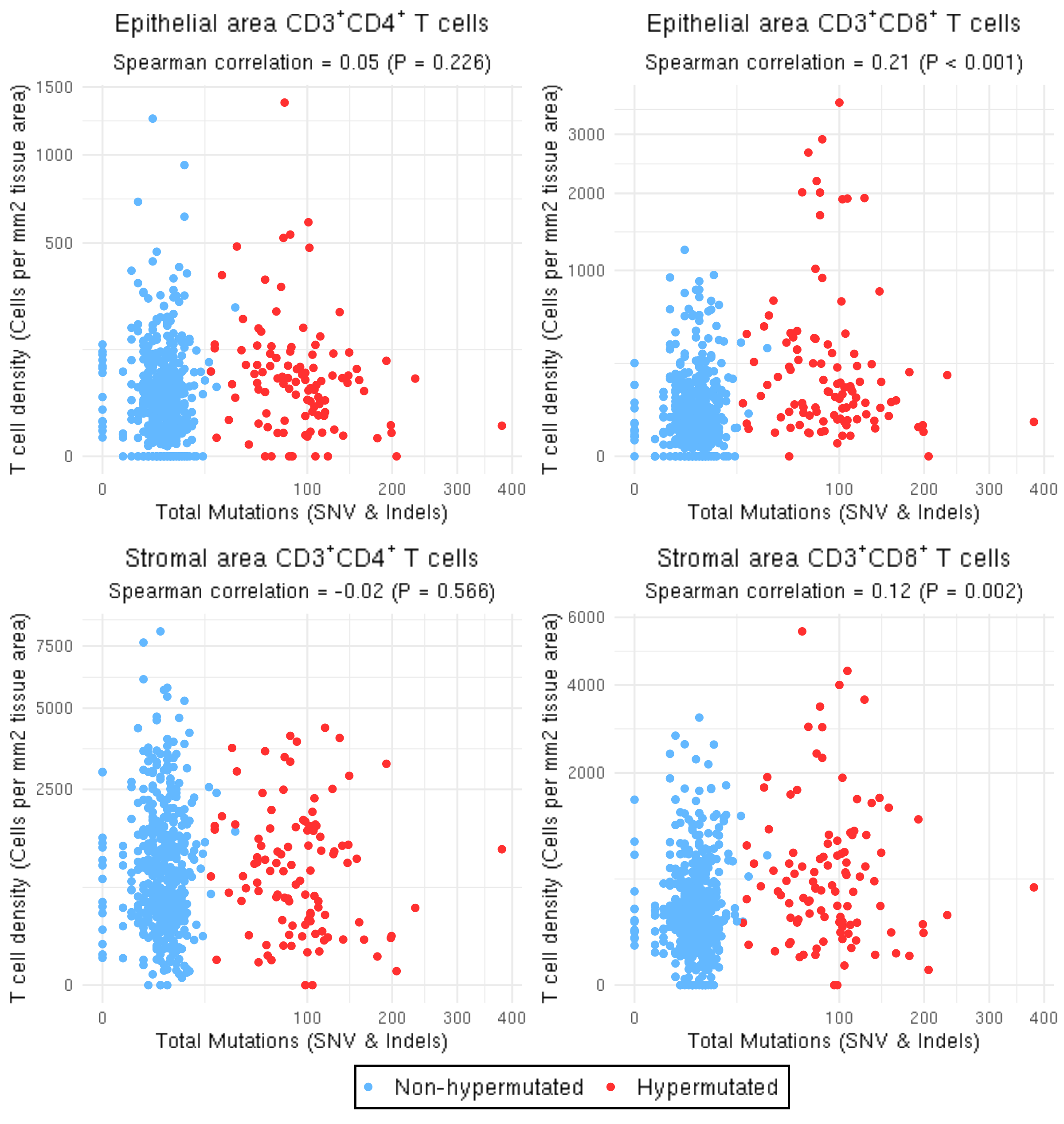

Supplement: Supplementary file 5 [file Image4.png]
